# Dissociation, trauma and the experience of visual hallucinations in post-traumatic stress disorder and schizophrenia

**DOI:** 10.1192/bjo.2023.3

**Published:** 2023-01-26

**Authors:** Yann Quidé

**Affiliations:** NeuroRecovery Research Hub, School of Psychology, UNSW Sydney, Sydney, NSW, Australia; and Centre for Pain IMPACT, Neuroscience Research Australia, Randwick, NSW, Australia

**Keywords:** Post-traumatic stress disorder, schizophrenia, trauma, hallucinations, dissociation

## Abstract

Dissociative behaviours and hallucinations are often reported in trauma-exposed people with schizophrenia spectrum disorders and post-traumatic stress disorder (PTSD). Auditory hallucinations are the most commonly reported type of hallucination, but often co-occur with experiences in other sensory modalities. The phenomenology and the neurobiological systems involved in visual experiences are not well characterised. Are these experiences similar in nature, content or severity among people with schizophrenia and/or PTSD? What are the neurobiological bases of these visual experiences and what is the role of dissociative behaviours in the formation of these experiences? A study by Wearne and colleagues in *BJPsych Open* aimed to characterise these phenomenological systems in groups of people with PTSD, schizophrenia or both (schizophrenia + PTSD).

Hallucinations are perceived as real sensory experiences occurring in absence of external stimuli, unlike other uncontrolled sensory phenomena (e.g. illusions). Although auditory and verbal hallucinations are the most commonly reported type of hallucination (around 10% in a lifetime),^1^ they often co-occur with phenomena from other sensory modalities (around 90% of the time).^2^ These phenomena are relatively common, especially during childhood and adolescence, but can become debilitating and pathological in psychotic disorders such as schizophrenia spectrum disorders.^3^ Prior exposure to childhood trauma (e.g. emotional, physical or sexual abuse, as well as emotional and physical neglect), a form of chronic stress with detrimental effects on the brain and on both physical and mental health, is commonly reported in people with psychotic disorders experiencing hallucinations (50–90%).^4^

Post-traumatic stress disorder (PTSD) is a debilitating psychiatric disorder that can occur following exposure to a traumatic event.^3^ PTSD is characterised by the avoidance of trauma reminders, hyperarousal, negative cognition and mood, and symptoms of intrusion of unwanted re-experiencing of the trauma, such as flashbacks or nightmares.^3^ In addition to these core symptoms, people suffering from PTSD can also experience dissociative behaviours, such as depersonalisation or derealisation.^5^ These dissociative behaviours are often used as a coping mechanism when exposed to traumatic events.^6^

## The relationship between trauma, PTSD and psychotic disorders

Experiences of dissociative behaviours largely overlap with the spectrum of psychotic experiences and have been associated with worse outcomes in PTSD.^5^ The relationship between trauma, PTSD and psychotic disorders has gained interest, with a diagnostic subtype of PTSD with psychotic features being proposed.^7^ Not surprisingly, people with PTSD, especially those reporting severe dissociative behaviours, also experience psychotic symptoms such as hallucinations.^8^ Dissociative behaviours can mediate the impact of trauma on the occurrence and content of hallucinations in people with a chronic schizophrenia spectrum disorder,^9^ but not in people with a first episode of psychosis.^10^ Another recent study also indicates that, although dissociation may mediate the impact of trauma on hearing voices, it does not explain all hallucinatory phenomena in trauma-exposed populations, with or without a diagnosis of schizophrenia spectrum disorder.^11^

Most studies have focused on the experience of auditory and verbal hallucinations in PTSD. These hallucinations have been reported in 20–58% of veterans with combat-related PTSD and in 67% of people developing PTSD following civilian trauma.^12^ Although auditory and verbal hallucinations reported in PTSD are mostly similar to those reported in schizophrenia,^13^ they can also differ in some ways, such as their presence, theme, nature, associated delusions, ego-syntonic characteristics and/or subjective distress.^14^ Less evidence is available on other hallucination modalities experienced by people with PTSD or schizophrenia. However, as hallucinations are likely to be multimodal psychotic features (predominantly auditory) reported by people with PTSD, and high rates of trauma exposure are reported by people with schizophrenia, one may speculate that hallucinations of other sensory modalities are likely to show similar patterns of similarities/differences to those observed with auditory hallucinations.

## Wearne and colleagues’ exploratory study

This was explored by Wearne and colleagues^15^ in the pages of *BJPsych Open*. The authors compared the phenomenology of visual hallucinations among three groups of individuals, all experiencing auditory hallucinations: a group with a diagnosis of schizophrenia, a group with a diagnosis of PTSD who all experienced dissociation and a group with diagnoses of both schizophrenia and PTSD (schizophrenia + PTSD). Importantly, there was no group difference in the phenomenology or the severity of visual hallucinations experienced, with people more likely to report experiences of complex visual hallucinations rather than illusions or simple hallucinations. These visual phenomena were reported as being real, irritating, distressing, difficult to ignore and associated with ‘acting out’ and fears of ‘losing one's mind’. Maybe not surprisingly, trauma-exposed groups (PTSD-only, schizophrenia + PTSD) were likely to report trauma-related hallucinations. Overall, this study provides preliminary evidence for similar rates, severity and nature of visual hallucinations across these diagnostic groups.

However, although associated with auditory hallucinations in all groups, dissociation was associated with visual hallucinations in the schizophrenia + PTSD group only. This group reported overall less severe PTSD and dissociative symptoms compared with the PTSD-only group. The authors proposed the possibility of two different types of visual phenomena in trauma-exposed populations: those present in flashbacks (re-experiencing the trauma, likely associated with experiencing peri-traumatic dissociative behaviours) and those related to chronic post-traumatic dissociative behaviours (trauma-related hallucinations). The differences between these two phenomena may be dependent on the severity of dissociative behaviours experienced and the engagement of the stress system in response to increasing threat.^16^ The dual representation theory^17^ indicates that peri-traumatic dissociation can affect the encoding of decontextualised memories, leading to the experience of flashbacks. On the other hand (chronic) post-traumatic dissociative behaviours are long-term experiences arising as a potential coping mechanism and occurring in presence of trauma-related stimuli reminding the victims of their traumatic experience. Although these two processes may involve different cognitive and neurobiological mechanisms, they are probably interdependent: visual hallucinations have been associated with the experience of peri-traumatic dissociation.^18^ According to the updated model of defence cascade,^19^ when lower levels of dissociation are experienced, heart rate reduces and the startle response is inhibited, making the individuals freeze in stupefaction. Visual phenomena involved in flashbacks may form as the severity of peri-traumatic dissociative behaviours increases. Increased release of cortisol and adrenaline via activation of the hypothalamic–pituitary–adrenal axis and the sympathetic nervous system respectively will increase heart rate and prepare the body in response to trauma exposure: the so-called ‘fight or flight’ response. When fleeing is not an option and as dissociation continues to increase, individuals can enter a phase of tonic immobility, the ‘fright’ phase, when both sympathetic and parasympathetic nervous systems are engaged. Long-term and chronic repetitions of these dissociative experiences, and associated changes in stress, sympathetic and parasympathetic functions, may generate more global and complex disturbances in perception, leading to the formation of visual hallucinations. Although the content and severity of the visual phenomena experienced are similar among trauma-exposed individuals with PTSD with or without schizophrenia, the biological processes and nature of these phenomena may be distinct, with severity and chronicity of dissociation indexing the type of visual phenomena experienced.

### Limitations and future research

Despite the important implications of these findings on the aetiology of these disorders, this exploratory study has important limitations. As noted by the authors, there is a need for replications in larger cohorts. In addition, all participants in this study reported experiencing auditory hallucinations. As hallucinations are often multimodal experiences, it is possible that the severity and/or content of the auditory hallucinations may have influenced the content, severity and/or occurrence of the visual phenomena. Future studies will need to determine the extent to which these auditory phenomena affect the perception of phenomena in other modalities in trauma-exposed populations. Finally, to confirm the proposed hypothesis of a separate type of visual phenomena, future neurobiological examinations are required to clearly establish the relationships between dissociation, the engagement of the sympathetic/parasympathetic nervous systems and the type of visual phenomena experienced. This knowledge is necessary to tailor personalised interventions specific to the type of visual phenomena experienced and will be critical in the definition and validity of a psychotic PTSD subtype. The present findings represent an excellent opportunity to call for the consistent recording of information about trauma exposure (in both childhood and adulthood), dissociation and psychotic features (e.g. hallucinations) in individuals with schizophrenia and/or PTSD. As the authors note, the development of a scale measuring visual experiences accessible to a large range of psychiatric disorders is warranted.

## Conclusions

The study by Wearne and colleagues^15^ is an important first step towards the definition of different visual experiences reported by trauma-exposed individuals with PTSD and/or schizophrenia. Different neurobiological systems may further characterise these types of visual experience, in the context of the severity of dissociation reported: flashbacks may be experienced when lower levels of peri-traumatic dissociation are reported, activating the sympathetic nervous system (‘fight or flight’), whereas hallucinations (multimodal, predominantly auditory plus visual) involving the parasympathetic nervous system (‘flag and faint’) are likely to form when more severe and chronic dissociative behaviours are reported. Future studies integrating phenomenology, neurobiology and neuroimaging are warranted to better understand and characterise these visual phenomena.

## Data Availability

Data availability is not applicable to this article as no new data was created or analysed in its preparation.
